# Supplementary material for: DNA transposon activity is associated with increased mutation rates in genes of rice and other grasses
Source: Nat Commun. 2016 Sep 7;7:12790. doi: 10.1038/ncomms12790 (PMC5023962; doi:10.1038/ncomms12790)
Supplement: Supplementary Information — Supplementary Figures 1 - 9, Supplementary Tables 1 - 5, Supplementary Notes 1 - 7 and Supplementary References [file ncomms12790-s1.pdf]

Supplementary Figures and Tables

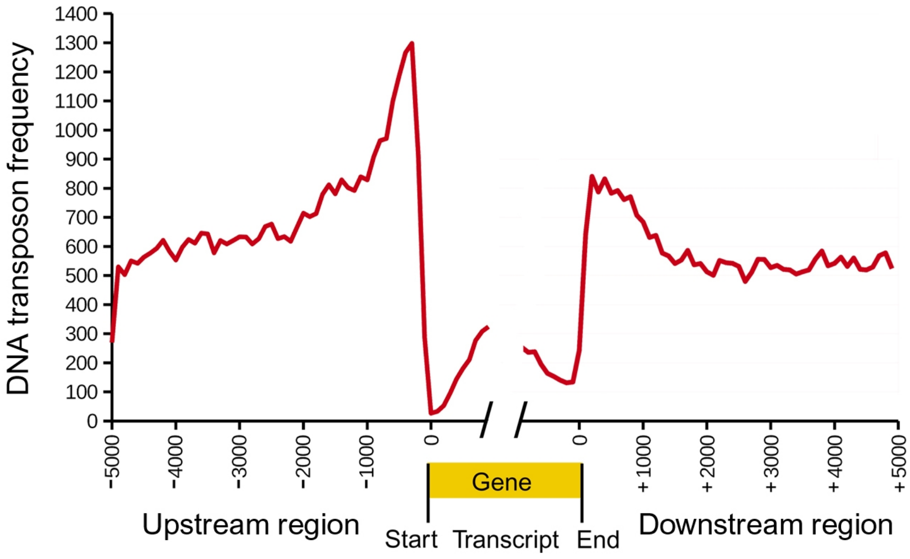

Supplementary Figure 1. Frequency of *DTT_Mariner* and *DTH_Harbinger* transposons relative to genes in *O. sativa*. Transposons in the up- and downstream regions of 21,444 genes were annotated and the cumulative occurrence plotted relative genes (e.g. the highest peak indicates that over 1,300 genes have a DNA transposon upstream of the transcription start point). The gene is shown in the center with 5,000 bp of up- and downstream region. Here, only genes longer than 2 kb were used. Thus, the center of the plot depicts the transposon frequencies of the 5' and 3' terminal 1,000 bp inside the genes.

Following page:

Supplementary Figure 2. Test for orthology for the loci containing putative transposon excisions. For this study, we manually identified 158 loci from rice chromosomes 1, 2 and 3 containing putative excisions of DNA transposons in either *O. sativa* or *O. glaberrima*. The Figure shows the positions of the compared loci on the *O. sativa* (Os) and *O. glaberrima* (Og) chromosomes. Putative orthologous loci are connected with blue lines. Loci are named after the gene closest to the polymorphic transposon. Since we aligned up to 24 kb of the putative orthologous loci, segments of 1 kb were used to map the genomic sequences back to the genomes (see methods). Each locus was assigned a score describing the percentage of 1 kb segments that mapped to its putative ortholog counterpart in the other species (orthology mapping score). The score is indicated as a small vertical box in the *O. sativa* chromosome. Obviously, some pf the 1 kb segments may map elsewhere in the genome because they are comprised of polymorphic TEs or repetitive sequences that can not be mapped unambiguously. However, most loci have very high scores, indicating that most parts of the 24 kb sequences of one species map unambiguously to the putative orthologous locus in the other species. Furthermore, all expect 2 loci are located in perfect colinear order along the chromosomes (see also methods).

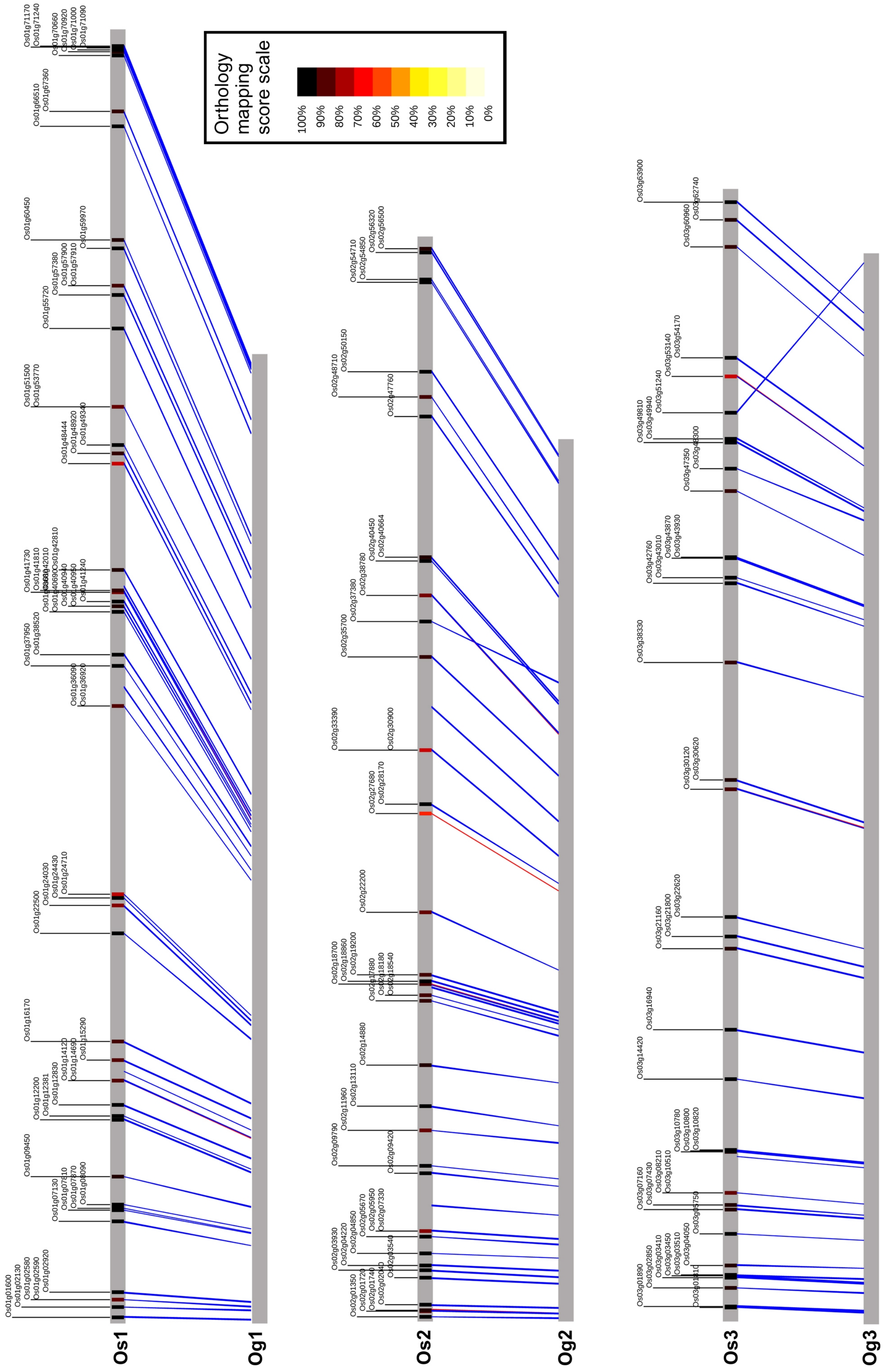
Os1
Og1
Os2
Og2
Os3
Og3
Orthology mapping score scale
100%
90%
80%
70%
60%
50%
40%
30%
20%
10%
0%
Os01g01600
Os01g02130
Os01g02580
Os01g02590
Os01g02920
Os01g07130
Os01g07810
Os01g07870
Os01g08090
Os01g09450
Os01g12200
Os01g12381
Os01g12830
Os01g14120
Os01g14690
Os01g15290
Os01g16170
Os01g22500
Os01g24030
Os01g24430
Os01g24710
Os01g36090
Os01g36920
Os01g37950
Os01g38520
Os01g41730
Os01g41810
Os01g42010
Os01g42810
Os01g40940
Os01g40950
Os01g41240
Os01g48444
Os01g48920
Os01g49340
Os01g51500
Os01g53770
Os01g55720
Os01g57380
Os01g57900
Os01g57910
Os01g59970
Os01g60450
Os01g66510
Os01g67360
Os01g71170
Os01g71240
Os01g70660
Os01g70920
Os01g71000
Os01g71090
Os02g01350
Os02g01720
Os02g01740
Os02g02040
Os02g03930
Os02g04220
Os02g04850
Os02g05670
Os02g05950
Os02g07330
Os02g03540
Os02g09790
Os02g09420
Os02g11960
Os02g13110
Os02g14880
Os02g18700
Os02g18860
Os02g19200
Os02g17880
Os02g18180
Os02g18540
Os02g22200
Os02g27680
Os02g28170
Os02g33390
Os02g30900
Os02g35700
Os02g37380
Os02g38780
Os02g40450
Os02g40664
Os02g48710
Os02g50150
Os02g47760
Os02g54710
Os02g54850
Os02g56320
Os02g56500
Os03g01890
Os03g02850
Os03g03410
Os03g03450
Os03g03510
Os03g04050
Os03g01810
Os03g05750
Os03g07160
Os03g07430
Os03g08210
Os03g10510
Os03g10780
Os03g10800
Os03g10820
Os03g14420
Os03g16940
Os03g21160
Os03g21800
Os03g22620
Os03g30120
Os03g30620
Os03g38330
Os03g42760
Os03g43010
Os03g43870
Os03g43930
Os03g47350
Os03g48300
Os03g49810
Os03g49940
Os03g51240
Os03g53140
Os03g54170
Os03g60960
Os03g62740
Os03g63900

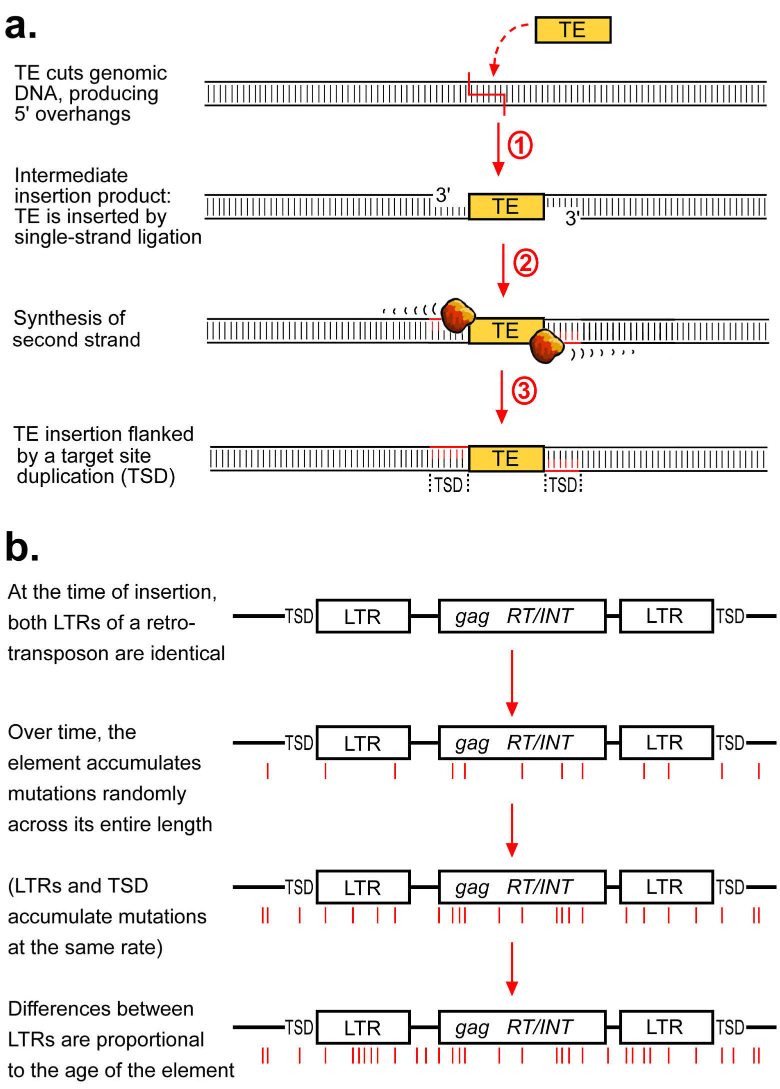

Supplementary Figure 3. Insertion and aging of transposable elements. (**a**) Model for the molecular mechanism of a typical TE insertion. Step 1: The TE inserts into the genome by producing a staggered cut, resulting in a TE that is ligated to the genomic DNA via single-stranded segments. Step 2. The single-stranded segments are filled by DNA polymerases. Step 3. The final outcome is the newly inserted TE that is flanked by a target site duplication (TSD). (**b**) Principle of how to estimate the age of a retrotransposon. Since TSD and LTRs are repeated sequences that were produced at the time of insertion, the number of differences accumulated are proportional to the age of the element. For plants, usually a mutation rate of 1.3 x 10^{-8} substitutions per site per year is used. However, if TSDs are produced by an error-prone polymerase complex, they tend to differ more strongly from each other than LTRs.

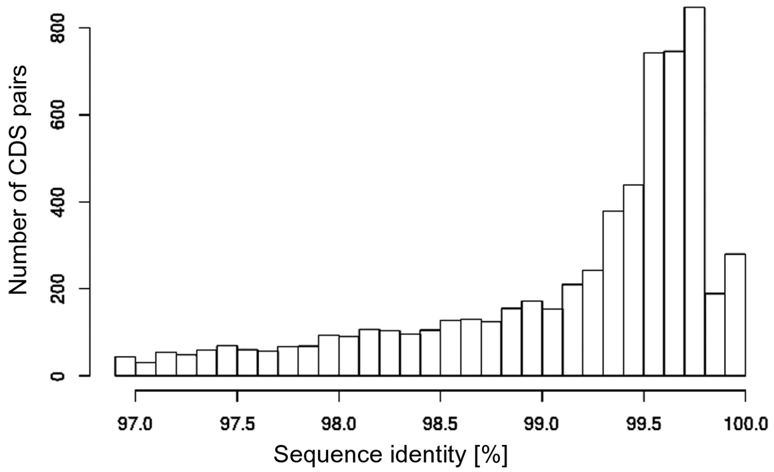

Supplementary Figure 4. Distribution of sequence identities of coding sequences (CDS) of closest homologs from *O. sativa* and *O. glaberrima*.

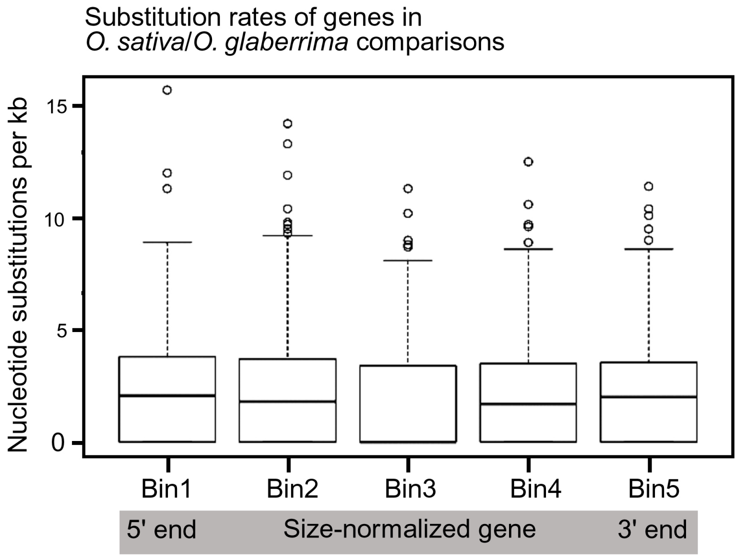

Supplementary Figure 5. Comparison of CDS of 312 genes from *O. sativa* and *O. glaberrima*. Here, only genes that are >99.5% identical (i.e. the overall level of sequence identity of the two genomes) were considered. The high conservation of these genes indicates that they were not affected by nearby error-prone DSB repair. They also do not show significantly lower sequence conservation in the center part of the gene.

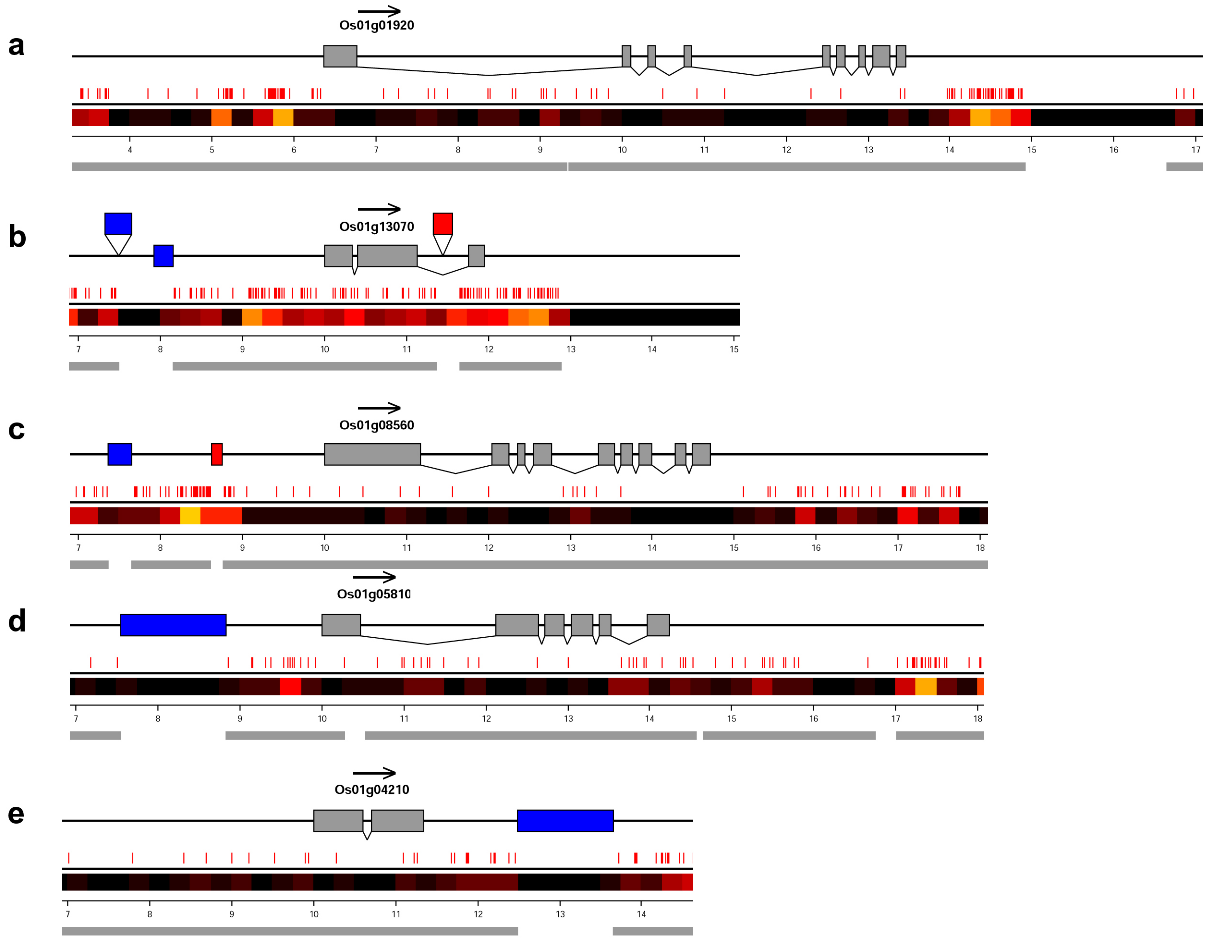

Supplementary Figure 6. Graphical representation of alignments of *O. sativa* and *O. glaberrima* genes plus 3 kb of their flanking regions (using start and end points of the predicted CDS as reference points). These were used for the identification of candidate regions that may contain TE excisions based on the number of sequence polymorphisms between *O. sativa* and *O. glaberrima*. Alignments of genes plus 3kb of their flanking sequences were analysed. The sequences of O. sativa were used as reference for the graphical display. Sequence annotation is shown at the top with exons of genes indicated as gray boxes. TE excisions are shown in red and TE insertions in blue. Inserted or excised TEs in *O. glaberrima* are depicted above the maps with lined pointing to the corresponding site in *O. sativa*. SNPs between *O. sativa* and *O. glaberrima* are indicated as red lines underneath the annotation. Underneath that track, SNP density is also visualized as a heat map in 25 bp windows. The gray bars at the bottom indicate the regions that could be aligned between *O. sativa* and *O. glaberrima.* (**a**-**c**) Examples for regions that where selected because they contain local SNP accumulations. (**d**) and (**e**) Examples for segments which served as controls and which have an overall low SNP density, similar to that of the genome-wide average.

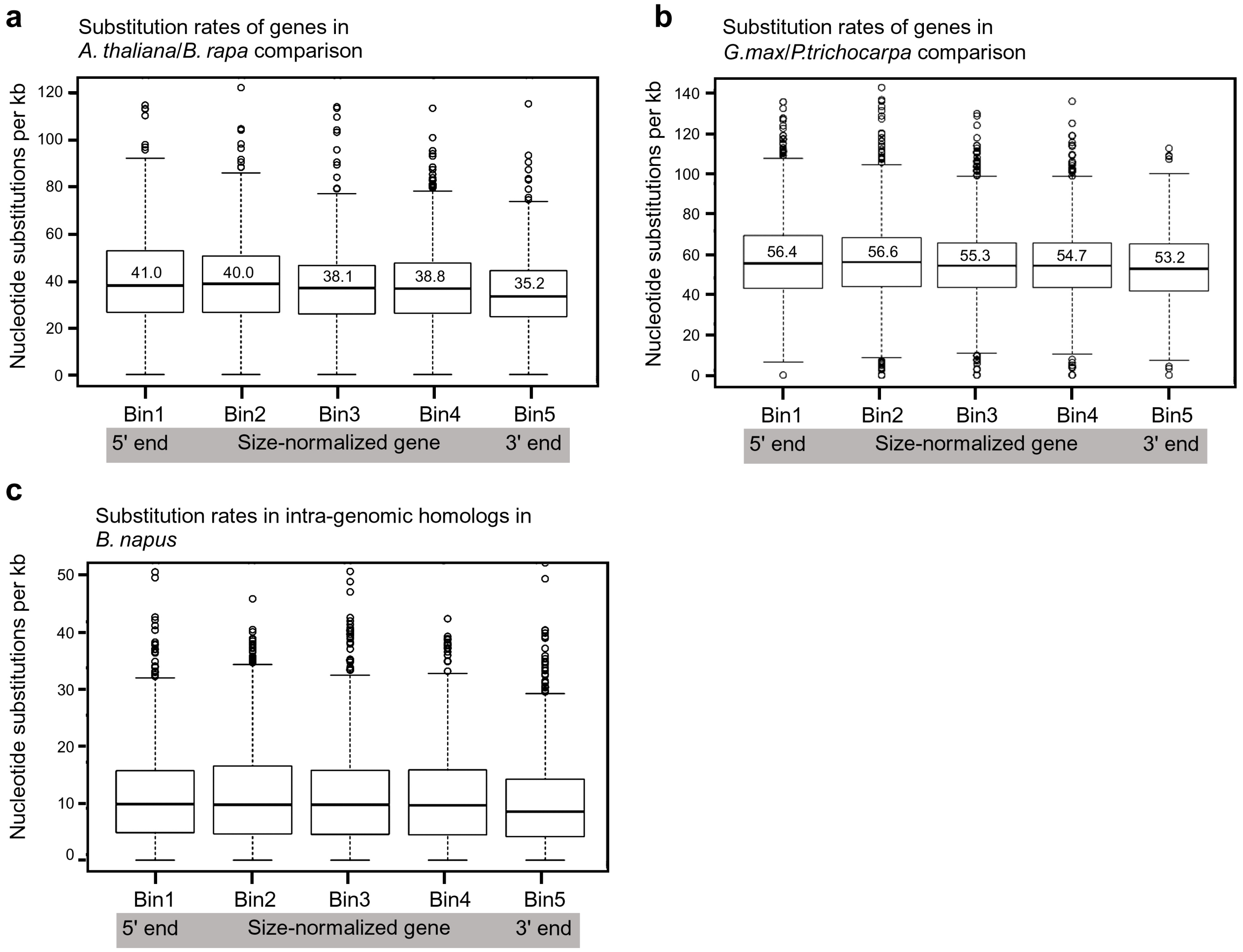

Supplementary Figure 7. Nucleotide substitution frequencies in synonymous sites of genes. To normalize the different CDS sizes, genes were divided into 5 equally sized bins and frequencies were normalized to nucleotide substitutions per kb for each bin. The bold line inside the box is the median value, while mean values are indicated with numbers. (**a**) Comparison of 636 pairs of closest homologs from *A. thaliana* and *B. rapa*. (**b**) Comparison of 1,799 pairs of closest homologs from soybean (*G. max*) and poplar (*P. trichocarpa*). (**c**) Nucleotide substitution frequencies in synonymous sites of of 1,395 pairs of intra-genomic closest homologs in *B. napus* that originate from a whole genome duplication.

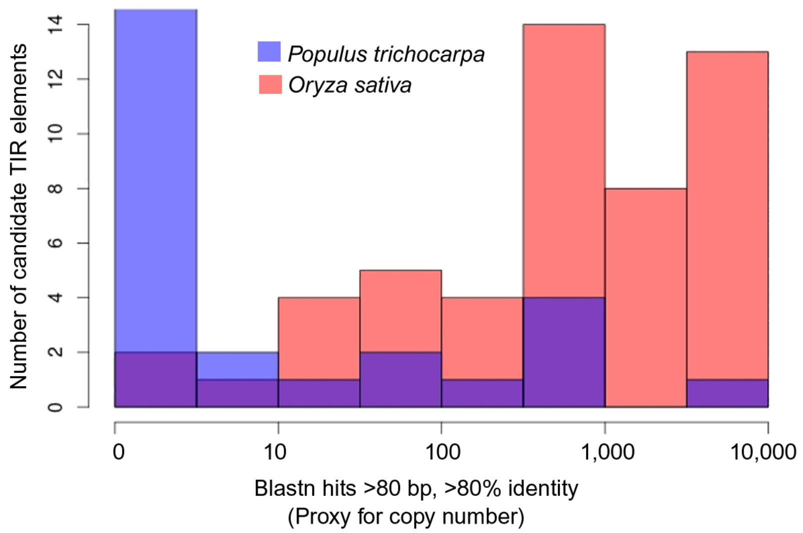

Supplementary Figure 8. Copy number estimates for candidate DNA transposons identified in *de novo* searches in the genomes of poplar (*P. trichocarpa*) and rice (*O. sativa*). As a proxy for copy numbers, each identified transposon candidates was used as a query in a blast search against its respective genome. All blast hits that were longer than 80 bp and >80% identical were considered. The x-axis shows the number of blast hits in a logarithmic scale while the y-axis shows the number of transposon candidates in each copy number range. Note that the *de novo* search in rice yielded many more elements which have on average much higher copy numbers than those in poplar.

a.

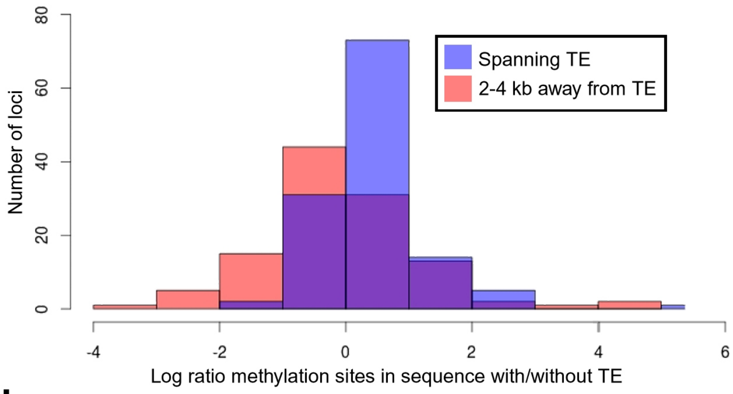

b.

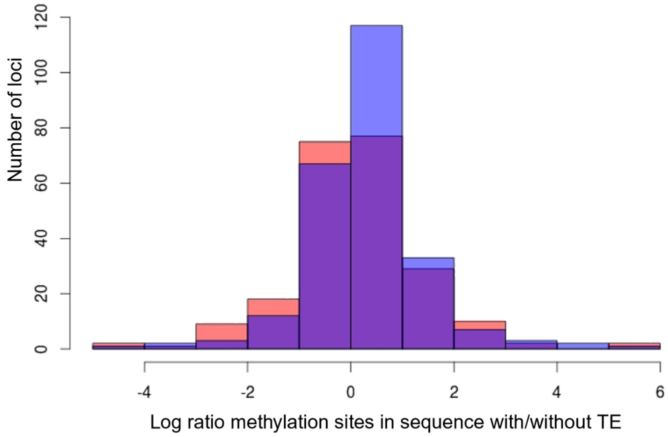

Supplementary Figure 9. Comparative analysis of methylation data in loci containing polymorphic transposons. Numbers of methylation sites were compared in orthologous loci with and without transposons in *O. sativa* and *O. glaberrima.* For each locus, the ratio of the numbers of methylated sites was calculated. The figure shows the distribution of the Log10 of these ratios. To study the effect of transposon insertions and excisions, data from 4 kb segments spanning the transposon (blue) site were compared with data from segments covering the sequence 2,000-4,000 bp away from the transposon (red). (**a**) Datasets for transposon excisions. (**b**) Datasets for transposon insertions. Note that in both datasets the ratio of numbers for sequence with transposon/sequence without transposon are shifted towards higher values, indicating that sequence segments containing transposons tend to have more methylated sites.

Supplementary Table 1. Positions of DNA (Class 2) transposon excisions in the two rice species *O. sativa* and *O. glaberrima*. Chromsomal positions are given for *O. sativa* genome version6 and *O. glaberrima* genome version 1. OsChr: *O. sativa* chromosome. OsPos: base pair position on *O. sativa* chromsome. OgChr: *O. glaberrima* chromosome. OgPos: base pair position on *O. glaberrima* chromsome.

OsChr	OsPos	OgChr	OgPos	Event
1	306959	1	211329	excision in *O. glaberrima*
1	616788	1	508545	excision in *O. sativa*
1	858433	1	627119	excision in *O. glaberrima*
1	864565	1	637088	excision in *O. glaberrima*
1	1074814	1	768963	excision in *O. glaberrima*
1	3360667	1	2590082	excision in *O. glaberrima*
1	3745430	1	2979321	excision in *O. sativa*
1	3803142	1	3006536	excision in *O. glaberrima*
1	3918145	1	3132783	excision in *O. glaberrima*
1	4815386	1	3847255	excision in *O. glaberrima*
1	6651580	1	4959979	excision in *O. sativa*
1	6765422	1	5047554	excision in *O. glaberrima*
1	7102225	1	5394206	excision in *O. sativa*
1	7925022	1	6070534	excision in *O. glaberrima*
1	8213940	1	6325790	excision in *O. sativa*
1	8553288	1	6685238	excision in *O. glaberrima*
1	9141251	1	7174597	excision in *O. sativa*
1	12649204	1	9251957	excision in *O. glaberrima*
1	13541075	1	9696587	excision in *O. sativa*
1	13772287	1	9835114	excision in *O. glaberrima*
1	13903805	1	10013470	excision in *O. sativa*
1	19973277	1	14353253	excision in *O. glaberrima*
1	20578900	1	14685479	excision in *O. sativa*
1	21259318	1	15128202	excision in *O. sativa*
1	21634661	1	15450764	excision in *O. glaberrima*
1	22993368	1	15917686	excision in *O. sativa*
1	22993368	1	15917686	excision in *O. sativa*
1	23164571	1	16062445	excision in *O. glaberrima*
1	23165619	1	16063174	excision in *O. glaberrima*
1	23342045	1	16158338	excision in *O. sativa*
1	23625935	1	16303696	excision in *O. glaberrima*
1	23675316	1	16441712	excision in *O. glaberrima*
1	23814701	1	16579729	excision in *O. sativa*
1	24364456	1	17122959	excision in *O. sativa*
1	27781873	1	19847466	excision in *O. glaberrima*
1	28072592	1	20070363	excision in *O. glaberrima*
1	28361571	1	20364679	excision in *O. sativa*
1	29591258	1	21479821	excision in *O. sativa*
1	29591258	1	21479821	excision in *O. sativa*
1	32090182	1	23123244	excision in *O. glaberrima*
1	33174763	1	24094927	excision in *O. sativa*
1	33480796	1	24350471	excision in *O. glaberrima*
1	33483408	1	24353462	excision in *O. sativa*
1	34672522	1	25183221	excision in *O. sativa*
1	34960832	1	25434965	excision in *O. sativa*
1	38626114	1	28741511	excision in *O. glaberrima*
1	39098738	1	29191544	excision in *O. glaberrima*
1	40901397	1	30670845	excision in *O. glaberrima*
1	41051298	1	30810778	excision in *O. sativa*
1	41093097	1	30853689	excision in *O. glaberrima*
1	41144155	1	30907102	excision in *O. glaberrima*
1	41177892	1	30952903	excision in *O. glaberrima*
1	41224131	1	31001343	excision in *O. glaberrima*
2	193005	2	144202	excision in *O. glaberrima*
2	400988	2	298559	excision in *O. sativa*

2	409744	2	310044	excision in *O. glaberrima*
2	570546	2	470835	excision in *O. sativa*
2	1441950	2	1258846	excision in *O. glaberrima*
2	1672674	2	1470629	excision in *O. glaberrima*
2	1852655	2	1673725	excision in *O. glaberrima*
2	2240965	2	2086762	excision in *O. sativa*
2	2774912	2	2521522	excision in *O. sativa*
2	2964408	2	2707426	excision in *O. glaberrima*
2	3778456	2	3461335	excision in *O. glaberrima*
2	4834349	2	4395923	excision in *O. glaberrima*
2	5051683	2	4634602	excision in *O. sativa*
2	6198543	2	5782266	excision in *O. sativa*
2	6963376	2	6351729	excision in *O. glaberrima*
2	8286770	2	7722270	excision in *O. sativa*
2	10361363	2	9244192	excision in *O. sativa*
2	10559040	2	9443107	excision in *O. sativa*
2	10805470	2	9624124	excision in *O. sativa*
2	10910114	2	9718146	excision in *O. glaberrima*
2	11008980	2	9839150	excision in *O. glaberrima*
2	11187934	2	9984296	excision in *O. glaberrima*
2	13233893	2	11353690	excision in *O. sativa*
2	16397439	2	13792955	excision in *O. sativa*
2	16672514	2	14149188	excision in *O. sativa*
2	18443613	2	15041579	excision in *O. sativa*
2	19833517	2	16144175	excision in *O. sativa*
2	21452668	2	17601177	excision in *O. sativa*
2	22574468	2	20611734	excision in *O. glaberrima*
2	23428543	2	18987091	excision in *O. sativa*
2	24523036	2	19921077	excision in *O. glaberrima*
2	24639439	2	20011778	excision in *O. glaberrima*
2	29194338	2	23380770	excision in *O. sativa*
2	29808797	2	23788631	excision in *O. glaberrima*
2	30632606	2	24572989	excision in *O. glaberrima*
2	33493057	2	27022104	excision in *O. sativa*
2	33582666	2	27103557	excision in *O. glaberrima*
2	34472769	2	27883966	excision in *O. sativa*
2	34589534	2	27943921	excision in *O. sativa*
3	499902	3	338565	excision in *O. sativa*
3	547017	3	384382	excision in *O. glaberrima*
3	1123914	3	964806	excision in *O. sativa*
3	1454688	3	1250217	excision in *O. glaberrima*
3	1491104	3	1284691	excision in *O. glaberrima*
3	1520100	3	1403811	excision in *O. glaberrima*
3	1858065	3	1754043	excision in *O. glaberrima*
3	2863754	3	2654354	excision in *O. glaberrima*
3	3661860	3	3356319	excision in *O. glaberrima*
3	3770950	3	3462134	excision in *O. glaberrima*
3	4185134	3	3828737	excision in *O. glaberrima*
3	5351516	3	5000021	excision in *O. glaberrima*
3	5505760	3	5126520	excision in *O. glaberrima*
3	5536148	3	5155184	excision in *O. glaberrima*
3	5551093	3	5170411	excision in *O. sativa*
3	7843648	3	7233157	excision in *O. glaberrima*
3	9419504	3	8701806	excision in *O. glaberrima*
3	12064524	3	11110004	excision in *O. sativa*
3	12455139	3	11463171	excision in *O. sativa*
3	13039003	3	12046638	excision in *O. glaberrima*
3	17195722	3	15931687	excision in *O. glaberrima*
3	17466945	3	16114828	excision in *O. sativa*
3	21272026	3	20148148	excision in *O. sativa*
3	23810131	3	22424470	excision in *O. glaberrima*
3	23988359	3	22633235	excision in *O. sativa*
3	24606514	3	23056031	excision in *O. sativa*

3	24647524	3	23102948	excision in *O. glaberrima*
3	26773320	3	24710738	excision in *O. glaberrima*
3	27496942	3	25840716	excision in *O. sativa*
3	28358918	3	26130190	excision in *O. sativa*
3	28466812	3	26233042	excision in *O. sativa*
3	29307053	3	34117698	excision in *O. glaberrima*
3	30468998	3	27578832	excision in *O. glaberrima*
3	31048314	3	28131446	excision in *O. sativa*
3	34631324	3	31127814	excision in *O. glaberrima*
3	35491552	3	31944763	excision in *O. sativa*
3	36097960	3	32506454	excision in *O. sativa*

Supplementary Table 2. Positions of DNA (Class 2) transposon insertions in the two rice species *O. sativa* and *O. glaberrima*. Chromsomal positions are given for *O. sativa* genome version6 and *O. glaberrima* genome version 1. OsChr: *O. sativa* chromosome. OsPos: base pair position on *O. sativa* chromsome. OgChr: *O. glaberrima* chromosome. OgPos: base pair position on *O. glaberrima* chromsome.

OsChr	OsPos	OgChr	OgPos	Event
2	548163	2	431986	insertion in *O. sativa*
2	1007456	2	841708	insertion in *O. glaberrima*
2	1097229	2	939142	insertion in *O. sativa*
2	1394657	2	1210000	insertion in *O. glaberrima*
2	1451345	2	1267611	insertion in *O. sativa*
2	1521432	2	1322748	insertion in *O. sativa*
2	3229543	2	2909625	insertion in *O. sativa*
2	3301024	2	2981497	insertion in *O. glaberrima*
2	3653146	2	3337718	insertion in *O. sativa*
2	3674570	2	3359044	insertion in *O. sativa*
2	3757652	2	3442306	insertion in *O. glaberrima*
2	3928217	2	3565077	insertion in *O. sativa*
2	4147768	2	3688881	insertion in *O. sativa*
2	4290583	2	3838942	insertion in *O. sativa*
2	4486929	2	4023272	insertion in *O. sativa*
2	4622678	2	4166409	insertion in *O. sativa*
2	4654407	2	4201648	insertion in *O. glaberrima*
2	4752142	2	4317340	insertion in *O. glaberrima*
2	5190270	2	4767482	insertion in *O. sativa*
2	5235725	2	4812481	insertion in *O. sativa*
2	5657121	2	5243037	insertion in *O. glaberrima*
2	5855629	2	5442276	insertion in *O. glaberrima*
2	5906514	2	5503460	insertion in *O. glaberrima*
2	5955309	2	5569589	insertion in *O. glaberrima*
2	6252471	2	5839700	insertion in *O. glaberrima*
2	6262767	2	5851858	insertion in *O. glaberrima*
2	6431234	2	6037740	insertion in *O. sativa*
2	6783920	2	6161665	insertion in *O. glaberrima*
2	6814392	2	6193919	insertion in *O. sativa*
2	6906056	2	6270107	insertion in *O. sativa*
2	7013533	2	6401988	insertion in *O. sativa*
2	7136689	2	6548048	insertion in *O. sativa*
2	7302742	2	6707731	insertion in *O. sativa*
2	7760791	2	7165360	insertion in *O. glaberrima*
2	8992287	2	8328170	insertion in *O. sativa*
2	9070594	2	8405295	insertion in *O. glaberrima*
2	9113359	2	8429490	insertion in *O. sativa*
2	9410673	2	8553323	insertion in *O. sativa*
2	10058277	2	9122738	insertion in *O. sativa*
2	10533680	2	9417561	insertion in *O. sativa*
2	10720163	2	9538327	insertion in *O. sativa*
2	10779807	2	9596088	insertion in *O. sativa*
2	10959876	2	9789025	insertion in *O. glaberrima*
2	11061761	2	9884599	insertion in *O. sativa*
2	14751202	2	12343679	insertion in *O. sativa*
2	15674238	2	13441149	insertion in *O. glaberrima*
2	16210731	2	13738559	insertion in *O. sativa*
2	17196696	2	14503969	insertion in *O. glaberrima*
2	17225368	2	14532168	insertion in *O. glaberrima*
2	18690505	2	15213556	insertion in *O. sativa*
2	18816844	2	15326737	insertion in *O. glaberrima*
2	19205531	2	15599243	insertion in *O. sativa*
2	19564156	2	15918400	insertion in *O. sativa*
2	19584120	2	15936738	insertion in *O. sativa*

2	19740850	2	16053640	insertion in *O. sativa*
2	19958660	2	16274099	insertion in *O. sativa*
2	20124694	2	16443706	insertion in *O. sativa*
2	20157721	2	16478652	insertion in *O. glaberrima*
2	20946541	2	17154650	insertion in *O. sativa*
2	21082955	2	17299069	insertion in *O. sativa*
2	21543303	2	17683708	insertion in *O. sativa*
2	21563217	2	17702980	insertion in *O. glaberrima*
2	21921457	2	17983530	insertion in *O. sativa*
2	22070941	2	18147353	insertion in *O. sativa*
2	22406944	2	18288259	insertion in *O. sativa*
2	22495996	2	22495996	insertion in *O. glaberrima*
2	22541034	2	20575505	insertion in *O. sativa*
2	22554806	2	20587784	insertion in *O. glaberrima*
2	22642951	2	18374193	insertion in *O. sativa*
2	22804060	2	18458294	insertion in *O. sativa*
2	22815346	2	18479046	insertion in *O. sativa*
2	23448710	2	18987738	insertion in *O. glaberrima*
2	23473847	2	19003942	insertion in *O. sativa*
2	23824527	2	19327044	insertion in *O. sativa*
2	23974169	2	19453644	insertion in *O. glaberrima*
2	24131443	2	19602959	insertion in *O. sativa*
2	24235675	2	19707384	insertion in *O. sativa*
2	24268744	2	19735158	insertion in *O. sativa*
2	24470903	2	19868817	insertion in *O. glaberrima*
2	24677182	2	20045537	insertion in *O. sativa*
2	25045807	2	20393831	insertion in *O. sativa*
2	25152424	2	20476874	insertion in *O. sativa*
2	25810130	2	20754995	insertion in *O. sativa*
2	26518050	2	21391569	insertion in *O. sativa*
2	27157357	2	21913411	insertion in *O. glaberrima*
2	27499344	2	22232412	insertion in *O. sativa*
2	27731114	2	22307406	insertion in *O. sativa*
2	28006147	2	22519911	insertion in *O. sativa*
2	28086917	2	22574545	insertion in *O. sativa*
2	28260263	2	22718849	insertion in *O. glaberrima*
2	28461725	2	22906233	insertion in *O. glaberrima*
2	28489288	2	22933156	insertion in *O. sativa*
2	29022471	2	23268211	insertion in *O. glaberrima*
2	29539680	2	23518558	insertion in *O. sativa*
2	29714180	2	23694148	insertion in *O. sativa*
2	29853203	2	23836420	insertion in *O. glaberrima*
2	30272346	2	24223963	insertion in *O. sativa*
2	30852647	2	24772078	insertion in *O. glaberrima*
2	32236122	2	25945231	insertion in *O. sativa*
2	32345377	2	26059054	insertion in *O. sativa*
2	32631080	2	26302291	insertion in *O. sativa*
2	32677692	2	26350340	insertion in *O. glaberrima*
2	32736887	2	26411599	insertion in *O. sativa*
2	32884148	2	26522127	insertion in *O. sativa*
2	33403287	2	26932179	insertion in *O. glaberrima*
2	33929106	2	27446500	insertion in *O. sativa*
2	34045288	2	27552982	insertion in *O. sativa*
2	34069760	2	27566980	insertion in *O. glaberrima*
2	34760092	2	28091069	insertion in *O. sativa*
2	34784954	2	28107797	insertion in *O. sativa*
2	34812529	2	28124520	insertion in *O. sativa*
2	35049936	2	28346461	insertion in *O. sativa*
2	35094170	2	28387094	insertion in *O. sativa*
2	35228196	2	28522776	insertion in *O. glaberrima*
2	35254201	2	28548082	insertion in *O. sativa*
2	35499374	2	28755864	insertion in *O. glaberrima*
2	35775697	2	28976705	insertion in *O. glaberrima*

3	179673	3	70226	insertion in *O. glaberrima*
3	432251	3	258020	insertion in *O. sativa*
3	483767	3	320333	insertion in *O. glaberrima*
3	603926	3	452443	insertion in *O. glaberrima*
3	742408	3	613190	insertion in *O. glaberrima*
3	1102395	3	944087	insertion in *O. sativa*
3	1241821	3	1074320	insertion in *O. sativa*
3	1277136	3	1097455	insertion in *O. sativa*
3	1300289	3	1117979	insertion in *O. sativa*
3	1532872	3	1416234	insertion in *O. sativa*
3	1784261	3	1666803	insertion in *O. sativa*
3	2229794	3	2097495	insertion in *O. sativa*
3	2419617	3	2241107	insertion in *O. sativa*
3	2459513	3	2275095	insertion in *O. sativa*
3	2749266	3	2545522	insertion in *O. sativa*
3	2810787	3	2602425	insertion in *O. glaberrima*
3	3177063	3	2921689	insertion in *O. glaberrima*
3	3272423	3	3009361	insertion in *O. glaberrima*
3	3493104	3	3209778	insertion in *O. sativa*
3	3753871	3	3445721	insertion in *O. sativa*
3	3792915	3	3481968	insertion in *O. glaberrima*
3	3831566	3	3520761	insertion in *O. sativa*
3	3967050	3	3618879	insertion in *O. sativa*
3	4136862	3	3784486	insertion in *O. sativa*
3	4853429	3	4488711	insertion in *O. sativa*
3	4907805	3	4548231	insertion in *O. sativa*
3	4945022	3	4584987	insertion in *O. sativa*
3	5632193	3	5250175	insertion in *O. sativa*
3	5655113	3	5271844	insertion in *O. glaberrima*
3	5714478	3	5335157	insertion in *O. sativa*
3	6153041	3	5776098	insertion in *O. sativa*
3	6951224	3	6390831	insertion in *O. sativa*
3	7006606	3	6450862	insertion in *O. glaberrima*
3	7093423	3	6537106	insertion in *O. glaberrima*
3	7194764	3	6615449	insertion in *O. sativa*
3	7229112	3	6649341	insertion in *O. glaberrima*
3	7594402	3	6991535	insertion in *O. glaberrima*
3	8097631	3	7487581	insertion in *O. sativa*
3	8350267	3	7723018	insertion in *O. glaberrima*
3	8506624	3	7861374	insertion in *O. sativa*
3	8597087	3	7940642	insertion in *O. glaberrima*
3	9013759	3	8330236	insertion in *O. sativa*
3	9154374	3	8450855	insertion in *O. sativa*
3	9216918	3	8500219	insertion in *O. glaberrima*
3	9356255	3	8640143	insertion in *O. glaberrima*
3	9505157	3	8786476	insertion in *O. sativa*
3	9739063	3	9012789	insertion in *O. sativa*
3	9964979	3	9241000	insertion in *O. sativa*
3	10107149	3	9298790	insertion in *O. sativa*
3	10234031	3	9421802	insertion in *O. sativa*
3	10419377	3	9611882	insertion in *O. sativa*
3	10945242	3	10103884	insertion in *O. glaberrima*
3	11254755	3	10315225	insertion in *O. sativa*
3	11480938	3	10512763	insertion in *O. glaberrima*
3	11512629	3	10555574	insertion in *O. sativa*
3	11583782	3	10631534	insertion in *O. glaberrima*
3	11721018	3	10778444	insertion in *O. sativa*
3	12737874	3	11759659	insertion in *O. sativa*
3	12760204	3	11774293	insertion in *O. sativa*
3	12805314	3	11811283	insertion in *O. sativa*
3	12938738	7	25264645	insertion in *O. sativa*
3	12960087	7	25421746	insertion in *O. glaberrima*
3	13005184	3	12012637	insertion in *O. sativa*

3	13260643	3	12272860	insertion in *O. sativa*
3	13700648	3	12572240	insertion in *O. sativa*
3	13712575	3	12584377	insertion in *O. glaberrima*
3	13829336	3	12706605	insertion in *O. sativa*
3	13884561	3	12746803	insertion in *O. sativa*
3	14292092	3	13096548	insertion in *O. sativa*
3	14467477	3	13356953	insertion in *O. sativa*
3	14519014	3	13411283	insertion in *O. sativa*
3	14728762	3	13543209	insertion in *O. glaberrima*
3	15651435	3	14362872	insertion in *O. sativa*
3	16059116	3	14827935	insertion in *O. sativa*
3	16155029	3	14922513	insertion in *O. sativa*
3	16857011	3	15661771	insertion in *O. sativa*
3	17050503	3	15820299	insertion in *O. glaberrima*
3	17522139	3	16160194	insertion in *O. sativa*
3	17911432	3	16575497	insertion in *O. glaberrima*
3	20141697	3	19009043	insertion in *O. sativa*
3	21004852	3	19895365	insertion in *O. glaberrima*
3	21147334	3	20033325	insertion in *O. sativa*
3	21198515	3	20084888	insertion in *O. sativa*
3	21228717	3	20116112	insertion in *O. sativa*
3	22848388	3	21603947	insertion in *O. sativa*
3	23158404	3	21856970	insertion in *O. sativa*
3	23277746	3	21956880	insertion in *O. sativa*
3	23470383	3	22125974	insertion in *O. glaberrima*
3	23505898	3	22158886	insertion in *O. sativa*
3	23542933	3	22193450	insertion in *O. sativa*
3	23562711	3	22214793	insertion in *O. glaberrima*
3	23610108	3	22252202	insertion in *O. sativa*
3	24220233	3	22709207	insertion in *O. glaberrima*
3	24498521	3	22935774	insertion in *O. glaberrima*
3	24731186	3	23190007	insertion in *O. sativa*
3	25272800	3	33592728	insertion in *O. glaberrima*
3	25539767	3	23581703	insertion in *O. sativa*
3	25596966	3	23638742	insertion in *O. sativa*
3	25898741	3	23829553	insertion in *O. sativa*
3	26014786	3	23946954	insertion in *O. sativa*
3	26572825	3	24531055	insertion in *O. glaberrima*
3	26698904	3	24647224	insertion in *O. glaberrima*
3	26815101	3	24751979	insertion in *O. glaberrima*
3	26870333	3	24806374	insertion in *O. sativa*
3	27707812	3	25229146	insertion in *O. glaberrima*
3	27778688	3	27778688	insertion in *O. sativa*
3	28130912	3	25635350	insertion in *O. sativa*
3	28261700	3	26033684	insertion in *O. sativa*
3	28641993	3	26403580	insertion in *O. glaberrima*
3	28684915	3	26447023	insertion in *O. glaberrima*
3	28755776	3	26519774	insertion in *O. glaberrima*
3	28829322	3	26593673	insertion in *O. glaberrima*
3	29142858	3	34306612	insertion in *O. sativa*
3	29668203	3	26839837	insertion in *O. sativa*
3	30095692	3	27217619	insertion in *O. sativa*
3	30871123	3	27970290	insertion in *O. sativa*
3	31279236	3	28302618	insertion in *O. glaberrima*
3	31346926	3	28370705	insertion in *O. sativa*
3	31919418	3	28912395	insertion in *O. sativa*
3	31953788	3	28948034	insertion in *O. glaberrima*
3	32042792	3	29037622	insertion in *O. sativa*
3	32081835	3	29071284	insertion in *O. sativa*
3	32615164	3	29533512	insertion in *O. sativa*
3	32657809	3	29575761	insertion in *O. sativa*
3	32685410	7	25338449	insertion in *O. glaberrima*
3	32850769	3	29675472	insertion in *O. sativa*

3	33498009	3	30112785	insertion in *O. glaberrima*
3	34260034	3	30761615	insertion in *O. sativa*
3	34327205	3	30828571	insertion in *O. sativa*
3	34770825	3	31278335	insertion in *O. sativa*
3	35092282	3	31559068	insertion in *O. glaberrima*
3	35436951	3	31885310	insertion in *O. sativa*
3	35732376	3	32150300	insertion in *O. glaberrima*
3	35806887	3	32222454	insertion in *O. sativa*

Supplementary Table 3. Substitution rates in target site duplications of long terminal repeat (LTR) retrotransposons compared to substitution rates in LTRs.

Family	Copies[a]	LTR [bp]	MM[c]	TSD[bp][d]	MM_{obs}[e]	MM_{exp}[f]	p-value
RLG_Hopi	82	87,857	369	410	20	1.7	>0.0001
RLG_Cara	36	19,455	674	175	18	6.1	>0.0001
RLC_Houba	74	69,487	977	365	13	5.1	0.0003
Total	192	176,799	2,020	950	51	10.9	>0.0001

[a]Number of full-length elements with intact ends that were flanked by a target site duplication (TSD)

[b]Total number of bases aligned between LTRs

[c]Number of mismatches in aligned LTR (bases for calculation of expected mismatches in TSDs.

[d]Total length in bp of aligned TSDs.

[e]Number observed of mismatches in aligned TSDs

[f]Number of mismatches expected in TSD based on substitution rates in LTRs

Supplementary Table 4. Wilcoxon rank sum test on comparisons of nucleotide substitutions within rice, barley, wheat, maize and Arabidopsis genes. To normalize for the different sizes of the genes, each gene was divided into 5 equally sized bins and nucleotide substitution frequencies were normalized to substitutions/kb for each bin. Given are the P-values for comparisons of data from all gene bins with all others. P-values smaller than 0.001 were considered significant (marked with *).

Bin pair	Os/Og[a]	Hv/Ta[b]	Maize (IG)[c]	At/Al[d]	Bn(IG)[e]	At/Br[f]	Gm/Pt[g]
1 vs. 2	0.002766	2.2E-16*	4.83E-09*	0.544	0.8738	0.7519	0.9398
1 vs. 3	4.702E-05*	2.2E-16*	5.553E-16*	0.02604	0.3248	0.06229	0.03457
1 vs. 4	0.00543	2.319E-14*	1.93E-08*	0.000138*	0.157	0.1195	0.00727
1 vs. 5	0.696	1.956E-05*	0.04769	1.614E-11*	6.262E-07*	4.733E-06*	1.67E-06
2 vs. 3	0.2863	0.002685	0.02406	0.00453	0.2357	0.1081	0.01868
2 vs. 4	0.7709	0.5643	0.7801	8.609E-06*	0.1153	0.2107	0.00395
2 vs. 5	0.008562	2.2E-16*	0.0002983*	1.518E-13*	7.45eE07*	1.604E-05*	4.75E-07
3 vs. 4	0.1702	0.0003636	0.01026	0.1264	0.6739	0.7326	0.5889
3 vs. 5	0.0002114*	2.2E-16*	6.578E-09*	1.431E-05*	1.219E-04*	0.004051	0.00591
4 vs. 5	0.01644	2.2E-16*	0.0007723*	0.004443	6.224E-04*	0.001096	0.026

[a]Comparison of 442 bi-directional closest homologs from *O. sativa* and *O. glaberrima.*

[b]Comparison of 2,314 bi-directional closest homologs from barley (*H. vulgare*) and wheat (*T. aestivum*)

[c]Comparison of 428 bi-directional closest homeologs within the maize genome that originated from a whole-genome duplication (WGD).

[d]Comparison of 4,133 bi-directional closest homologs from *A. thaliana* and *A. lyrata*.

[e]Comparison of 1,395 bi-directional closest homeologs within the *Brassica napus* genome that originated from a WGD.

[f]Comparison of 536 bi-directional closest homologs from *A. thaliana* and *B. rapa* (the A genome of B. napus)

[g]Comparison of 1,799 bi-directional closest homologs from *Glycine max* and *Populus trichocarpa.*

Supplementary Table 5. Datasets of coding regions (CDS) used for comparative Analyses.

Species	genome version	source
Arabidopsis thaliana	9	arabidopsis.org
Arabidopsis lyrata	1.0	genome.jgi-psf.org/Araly1
Brassica napus	5	brassicadb.org/brad
Brassica rapa	1.5	brassicadb.org/brad
Glycine max	1	plantgdb.org/GmGDB
Hordeum vulgare	1.1	pgsb.helmholtz-muenchen.de/plant
Oryza sativa	6	plantgdb.org/OsGDB
Oryza glaberrima	1.0	genome.arizona.edu
Populus trichocarpa	2.2	plantgdb.org/PtGDB
Triticum aestivum	2.2	pgsb.helmholtz-muenchen.de/plant
Zea mays	1.0	maizegdb.org

Supplementary Notes

Contents

1. Transposable elements and their contribution to evolution

2. Background on grass comparative genomics

3. Methodological considerations on distinguishing transposon excisions from insertions

4. Test for orthology of compared sequence segments

5. Brassicaceae do not show increased mutation rates in termini of genes

6. Comparative analysis of methylation states in polymorphic transposon loci

7. Evaluation of evidence for transposons as the cause for increased mutation rates in genes

Supplementary Note 1: Transposable elements and their contribution to evolution

DNA transposons can excise from the genome and re-insert elsewhere. When transposons excise, they leave double-strand breaks (DSBs) that have to be repaired by the cell. Depending on the repair pathway, this can lead to deletions and/or insertions of “filler” sequences at the site of the DSB (1-3).

In most eukaryotes, non-homologous end joining (NHEJ) is the main pathway for DSB repair. Here, the broken ends are directly ligate. However, other pathways are more complex, and include single-stranded intermediates. Here, the initial step in DSB repair is the generation of 3' overhangs through exonucleases at the site of the break. Depending on the time that elapses before other repair enzymes are recruited, these 3' overhangs can be several kb in size, at least in yeast (4). The 3' overhangs can directly anneal to each other by single-strand annealing (SSA), using a few bp of micro-homology (reviewed by 5,6). This ultimately leads to a deletion of the segment between the annealing motifs. Previous studies showed that such deletions can range from a few bp (1,3) to several kb (2,3). Alternatively, a 3' overhang can invade a foreign DNA strand and use it as an intermediate template for DNA synthesis in a process called synthesis-dependent strand annealing (5-7). This leads to the introduction of a copy of the foreign

template at the DSB site. Repair is completed when the leftover single-stranded DNA segments are used as templates for the synthesis of a new second strand. Sometimes, deletions and filler insertions at the excision site can be so extensive that transposon excisions are very difficult to identify as such, thereby explaining the generally low number of identified excisions (2,3).

How much transposable elements (TEs) contribute to the evolution of genes and species is still unclear. Certainly, there have been cases where TEs contributed to major evolutionary innovations. For example the V(D)J recombination in the vertebrate immune system most likely has its origin in a transposable element (8). Additionally, there have been several studies showing that TEs can generate novel genic sequences, for example through gene retrotransposition or by providing new exons in a process called exonization (9). There are also many studies that described their influence on gene expression (example in 10). Thus, evidence for TE-driven evolutionary innovation is patchy and often anecdotal and the quantitative contribution of TEs to genome evolution is still unknown (9,10).

Supplementary Note 2: Background on grass comparative genomics

Grasses evolved from a common ancestor approximately 70 Myr ago (11). They are part of the major plant group of the monocotyledons which diverged from its "sister" group, the dicotyledons, approximately 145-300 Myr ago (12,13). Grasses provide an excellent dataset for comparative analyses because the genomes of representatives of the major clades *Bambusoideae*, *Ehrhartoideae* and *Pooideae* have been sequenced. This allows comparative analyses between clades, for example between the genomes of rice (14) and maize (15) as well as within clades, for example of wheat (16) and barley (17).

Most DNA transposons described to date in grasses are small non-autonomous derivatives which do not encode any proteins and which depend for their transposition on transposase enzymes that are encoded by a small number of autonomous "mother" elements (18,19). Some of the non-autonomous elements (mostly those of the *DTT_Mariner* and *DTH_Harbinger* superfamilies) are referred to as miniature

inverted-repeat transposable elements (MITEs, 20,21). Due to their small size they only contribute relatively little to the overall genome size and often seem to be tolerated in or near genes (18,20,21).

Supplementary Note 3: Methodological considerations on distinguishing transposon excisions from insertions

It is surprisingly difficult to identify transposon excision events in a comparative analysis. It was therefore essential to our study that we could distinguish transposon excisions from insertions with high confidence. We defined stringent criteria for an event to be classified as an excision, and preferred to discard unclear events. Previous studies showed that transposons excisions can produce a variety of patterns, including deletions and insertions of filler sequences (1,2,3,22). Since deletions and filler insertions can obscure excisions beyond recognition, or because deletions could by chance remove entire transposons, we required that at least one breakpoint of the deletion of filler insertion be within 3 bp of one end of the transposon (we considered it unlikely that a random deletion would have one of its borders so close to the end of a TE).

Furthermore, it is possible that some events we classified as insertions are in fact excisions that removed the transposon and precisely one copy of the target site. Such events were defined as "precise" excisions by Yang et al. (22). In a comparative analysis such as ours, it is impossible to distinguish precise excisions from insertion events. Interestingly, there are conflicting reports on the frequency of precise excisions. Using a heterologous system expressing the rice mPing element in Arabidposi, Yang et al. (22) reported that 25 of 30 excisions were precise. In contrast, Kikuchi et al. (23), working with the same element in rice anther cultures, found only one out of approximately 70 excision events to be precise. Also our own data suggest that the proportion of precise excisions may be small: we compared transposon polymorphisms which we classified as insertions with insertions of *Gypsy* retrotransposons (which can not excise). Both show similar increased mutation frequencies in their flanking regions, indicating that insertions also induce mutations in nearby sequences (which is not surprising, since the

insertion process also has single-stranded intermediates). Nevertheless, insertions show overall much fewer mutations in their flanking regions than events that were classified as excisions (see Figure 3; Supplementary Figure 3). From this, we conclude that our criteria indeed distinguish different types of events (i.e. excisions and insertions) and that the events we classified as insertions contain only few precise excisions.

Supplementary Note 4: Test for orthology of compared sequence segments

Because we make a major claim about the role of TEs in evolution, it is important that concerns over potential weaknesses are addressed in detail. Thus, critical factors in our methods as well as in the interpretation of the results are discussed in the following. A crucial part of our case was to make sure that indeed orthologous loci were compared. Otherwise one could argue that putative excision sites that contain many polymorphisms are simply distant paralogs of which one never actually contained a transposon. Independent mapping of the analyzed sequences back onto the genomes showed that the analyzed loci all have exactly one homolog in each of the species, with almost all putative orthologs being located in colinear positions along chromosomes (Supplementary Fig 2). Theoretically, there is also the possibility that we compare deep paralogs, where a duplicated locus was present in the rice ancestor and subsequently, one copy was deleted in one species while the second copy was deleted in the other. This is a well-known problem in multi-copy gene families (example in 24). But sequence homology of such deep paralogs usually does not extend much past the sequences of the affected genes, while we aligned segments of up to 24 kb in size. We are thus confident that the vast majority of the sequences analyzed indeed represent orthologous loci.

Supplementary Note 5: Brassicaceae do not show increased mutation rates in termini of genes

To study whether the impact of DNA transposons is a general phenomenon in plants, we compared

closest gene homologs in representatives of the dicotyledons which diverged from the monocotyledons about 145-300 Myr ago (12,13). We used multiple dicotyledon species, representing major lineages as well as different degrees of evolutionary distance. *Brassica rapa*, *B. napus*, *Arabidopsis thaliana* and *A. lyrata* were chosen as representatives of the *Brassicaceae* family. *A. thaliana* and *A. lyrata* diverged from each other approximately 10 Myr ago (see methods) while *Brassica* and *Arabidopsis* diverged approximately 32 Myr ago. Poplar (*Populus trichocarpa*) and soybean (*Glycine max*), which diverged approximately 70 Myr ago, were chosen as representatives of the *Fabid* clade. Interestingly, in none of the comparisons did we find increased substitution rates in terminal regions of genes (Figure 4D, Supplementary Figure 6, Supplementary Table 5), suggesting that there is no effect of DNA transposons on genes comparable to that found in grasses.

Since we found a strong association of mutation rates in grass genes with DNA transposons activity, we expected that the genomes of dicotyledons contain fewer such elements. Therefore, we performed a *de novo* search for DNA transposons in the *A. thaliana* genome (see methods), in order to assess the abundance of these elements. Interestingly, we found only 27 different types of putative transposons, which were present in a total of 330 copies in *A. thaliana*. Furthermore, many of these elements are only fragments, as we classified only 65 as potentially intact elements. Thus, *A. thaliana* contains several orders of magnitude fewer DNA transposons than the grass genomes sequenced so far [8,28]. We also performed the *de novo* search in the *P. trichocarpa* genome which is with 495 Mbp even larger than the *O. sativa* genome. Here, we manually examined all 31 candidate transposons that were identified in the first 2 Mbp of linkage group 1. Only two turned out to be DNA transposons that are present at moderately high copy numbers (approximately 450 and 600 copies, respectively). In contrast, the same *de novo* search in only 500 kb in rice yielded 53 candidates, of which 20 had over 500 copies in the genome (Supplementary Figure 7).

Supplementary Note 6: Comparative analysis of methylation states in polymorphic transposon

loci

To study whether transposon excisions and insertions have an effect on the methylation state of the respective locus, we compared methylation data from *O. sativa* and *O. glaberrima* (see methods). Sequence segments of 4 kb spanning the polymorphic transposon in *O. sativa* and *O. glaberrima* were extracted from the chromosomes. The sequences were the aligned and positions of methylated bases compared. We we found that practically no methylation sites were conserved between the two species. Thus, overall methylation states were compared by simply counting the numbers of methylated sites in the sequences segments from the two species. The ratio of the number of methylation sites in *O. sativa* and *O. glaberrima* was then calculated for each transposon locus. For comparison, a second segment 2,000-4,000 bp downstream of the transposon was extracted. For excisions, we found a weak but significant (Wicoxon test p-value = 3.893e-05) difference in the two distributions (Supplementary Fig. 8). These data suggest that transposon excisions tend to be followed by de-methylation of the locus. For insertions the effect was weaker but still statistically significant (Wicoxon test p-value =0.008, Supplementary Fig. 8b). However, since practically no methylated sites were conserved in the two species and the loci studied, the described quantitative analysis is crude and we do not want to over-interpret these results.

Supplementary Note 7: Evaluation of evidence for transposons as the cause for increased mutation rates

Obviously, there are other possible causes for DSBs near genes besides transposon excisions, such as toxic chemicals, radiation or template breakage or slippage during replication. Following the repair pathway described in Figure 3, this could also lead to mutations during DSB repair. However, several lines of evidence support our claim that DNA transposons are at least a major factor leading to the elevated mutation rates in CDS and regulatory regions in grasses. First, our data from sequence comparisons show empirically that sequences flanking excisions contain highly elevated numbers of

nucleotide substitutions and InDels. Since DNA transposons are strongly enriched in promoter and downstream regions, it follows that these regions will be disproportionately affected. We indeed find that promoters are one average less conserved than randomly picked intergenic sequences. Second, genes from *O. sativa* and *O. glaberrima* which have the highest sequence conservation, reflecting the overall genome-wide average, do not show a substitution rate gradient. In contrast, genes that have a below average sequence conservation show the gradient. Third, genomes which contain many DNA transposons (such as grasses) all show the substitution rate gradient in genes, while those of dicotyledons (which contain much fewer DNA transposons) do not.

Supplementary References

1. Yang G., Weil CF. and Wessler SR. A rice Tc1/mariner-like element transposes in yeast. Plant Cell. 2006; **18:** 2469–2478.
2. Buchmann JP, Matsumoto T, Stein N, Keller B, Wicker T. Interspecies sequence comparison of Brachypodium reveals how transposon activity corrodes genome colinearity. Plant J. 2012; **488:** 213-217.
3. Roffler S, Wicker T. 2015. Genome-wide comparison of Asian and African rice reveals high recent activity of DNA transposons. Mob DNA. 2015; **6:**8.
4. Storici F, Snipe JR, Chan GK., Gordenin, D.A. Resnick, M.A. Conservative repair of a chromosomal double-strand break by single-strand DNA through two steps of annealing. J Cell Biol. 2006; **26:** 7645-7657.
5. Puchta, H. The repair of double-strand breaks in plants: Mechanisms and consequences for genome evolution. J Exp Botany. 2005; **56:** 1–14.
6. Hartlerode AJ, Scully R. Mechanisms of double-strand break repair in somatic mammalian cells. Biochem J. 2009; **423:** 157–168.
7. Nassif N, Penney J, Pal S, Engels WR, Gloor GB. Efficient copying of nonhomologous sequences from ectopic sites via P-element-induced gap repair. Mol Cell Biol 1994; **14:** 1613–1625.
8. Fugmann SD, Lee AI, Shockett PE, Villey IJ, Schatz DG. The RAG proteins and V(D)J recombination: complexes, ends, and transposition. Ann Rev Immunol. 2000; **18:** 495-527.
9. Cordaux R, Batzer MA. The impact of retrotransposons on human genome evolution. Nat Rev Genet. 2009; **10:** 691-703.
10. de Souza, F.S., Franchini, L.F. Rubinstein, M. Exaptation of transposable elements into novel cis-regulatory elements: is the evidence always strong? Mol Biol Evol. 2013; **30:** 1239-1251.
11. Grass Phylogeny Working Group. Phylogeny and subfamilial classification of the grasses (Poaceae). Ann Mo Bot Gard. 2001; **88:** 373–457.

12. Kawai Y, Otsuka J. The deep phylogeny of land plants inferred from a full analysis of nucleotide base changes in terms of mutation and selection. J Mol Evol. 2004; **58:** 479–489.

13. Zimmer, A, Lang, D, Richardt, S, Frank, W, Reski, R and Rensing, SA. Dating the early evolution of plants: detection and molecular clock analyses of orthologs. Mol Gen Genomics. 2007; **278:** 393–402.

14. International Rice Genome Sequencing Project. The map-based sequence of the rice genome. Nature. 2005; **436:** 793–800.

15. Schnable PS, Ware D, Fulton RS, Stein JC, Wei F. et al. The B73 maize genome: complexity, diversity, and dynamics. Science. 2009; **326:** 1112-1115.

16. International Wheat Genome Sequencing Consortium (IWGSC). A chromosome-based draft sequence of the hexaploid bread wheat (Triticum aestivum) genome. Science. 2014; **345:** 1251.

17. International Barley Genome Sequencing Consortium (IBSC), Mayer KF, Waugh R, Brown JW, Schulman AH et al. A physical, genetic and functional sequence assembly of the barley genome. Nature. 2012; **491:** 711-716.

18. International *Brachypodium* Initiative. Genome sequencing and analysis of the model grass *Brachypodium distachyon*. Nature. 2010; **463:** 763–768.

19. Yang G., Weil CF. and Wessler SR. A rice Tc1/mariner-like element transposes in yeast. Plant Cell. 2006; **18:** 2469–2478.

20. Bureau T, Wessler SR. Mobile inverted-repeat elements of the Tourist family are associated with the genes of many cereal grasses. Proc Natl Acad Sci USA 1994; **9:** 907-916.

21. Bureau T, Wessler SR. Stowaway: a new family of inverted repeat elements associated with the genes of both monocotyledonous and dicotyledonous plants. Proc Natl Acad Sci USA 1994; **9:** 1411-1115.

22. Yang G, Zhang F, Hancock CN, Wessler SR. Transposition of the rice miniature inverted repeat transposable element mPing in Arabidopsis thaliana. Proc Natl Acad Sci USA. 2007; 104:10962-10967.

23 .Kikuchi K, Terauchi K, Wada M, Hirano HY. The plant MITE mPing is mobilized in anther culture. Nature. 2003; 421:167-70.

24. Bossolini E, Wicker T, Knobel PA, Keller B. Comparison of orthologous loci from small grass genomes Brachypodium and rice: implications for wheat genomics and grass genome annotation. Plant J. 2007; 49:704-717.
